# Diabetes‐related clinical and microstructural white matter changes in patients with Alzheimer's disease

**DOI:** 10.1002/brb3.3533

**Published:** 2024-05-07

**Authors:** Betul Sumbul‐Sekerci, Halil Aziz Velioglu, Abdusselam Sekerci

**Affiliations:** ^1^ Department of Clinical Pharmacy, Faculty of Pharmacy Bezmialem Vakıf University Istanbul Turkey; ^2^ Center for Psychiatric Neuroscience Feinstein Institutes for Medical Research Manhasset New York USA; ^3^ Department of Neuroscience, Faculty of Medicine Istanbul Medipol University Istanbul Turkey; ^4^ Department of Internal Medicine, Faculty of Medicine Bezmialem Vakif University Istanbul Turkey

**Keywords:** Alzheimer's disease, cognitive impairment, diabetes, diffusion tensor imaging, nephropathy

## Abstract

**Aim:**

Although there exists substantial epidemiological evidence indicating an elevated risk of dementia in individuals with diabetes, our understanding of the neuropathological underpinnings of the association between Type‐2 diabetes mellitus (T2DM) and Alzheimer's disease (AD) remains unclear. This study aims to unveil the microstructural brain changes associated with T2DM in AD and identify the clinical variables contributing to these changes.

**Methods:**

In this retrospective study involving 64 patients with AD, 31 individuals had concurrent T2DM. The study involved a comparative analysis of diffusion tensor imaging (DTI) images and clinical features between patients with and without T2DM. The FSL FMRIB software library was used for comprehensive preprocessing and tractography analysis of DTI data. After eddy current correction, the “bedpost” model was utilized to model diffusion parameters. Linear regression analysis with a stepwise method was used to predict the clinical variables that could lead to microstructural white matter changes.

**Results:**

We observed a significant impairment in the left superior longitudinal fasciculus (SLF) among patients with AD who also had T2DM. This impairment in patients with AD and T2DM was associated with an elevation in creatine levels.

**Conclusion:**

The white matter microstructure in the left SLF appears to be sensitive to the impairment of kidney function associated with T2DM in patients with AD. The emergence of AD in association with T2DM may be driven by mechanisms distinct from the typical AD pathology. Compromised renal function in AD could potentially contribute to impaired white matter integrity.

## Graphical abstract

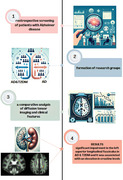

## 1 INTRODUCTION

The human brain, being highly metabolically active, is susceptible to the impact of glucose metabolism dysregulation, a prominent characteristic of diabetes. In addition to the various known micro‐ and macrovascular complications of diabetes, impaired glucose metabolism is likely to have adverse consequences on neural functioning and cognitive processes (Liu et al., 2019). The association between cognitive impairment and Type‐2 diabetes mellitus (T2DM) is gaining recognition as a significant comorbidity and complication (Koekkoek et al., 2015). An extensive quantitative meta‐analysis conducted revealed that diabetes increased the risk of incident Alzheimer's disease (AD), vascular dementia, and mild cognitive impairment (Cheng et al., 2012).

Previous studies have suggested that the overlapping mechanisms and risk factors between aging and Alzheimer's with T2DM. AD and T2DM may represent a spectrum of cognitive impairment marked by accelerated aging processes (Biessels & Reijmer, 2014; Exalto et al., 2012). Although there is compelling epidemiological evidence linking diabetes to a higher risk of dementia, it does not seem to result in an increased neuropathology of AD in T2DM (Abner et al., 2016; Matioli et al., 2017). However, the exact underlying mechanisms and causality linking diabetes to specific cerebral changes remain largely elusive (Biessels & Whitmer, 2020). The effect of glucose metabolism disruptions in diabetes on brain functions and microstructure attracts the attention of neuroimaging studies. Individuals diagnosed with T2DM exhibited irregularities in the microstructure of different white matter pathways, which were found to be associated with impaired cognitive performance (Reijmer et al., 2013). AD and T2DM are both considered to be related to reduced brain volumes, microstructural abnormalities. Nevertheless, the association between hyperglycemia and microstructural changes in AD patients remains unclear.

Diffusion tensor imaging (DTI) is an advanced neuroimaging technique that provides detailed insights into the microstructural organization of brain tissue, especially the white matter tracts. This method is pivotal in characterizing the microstructural changes or differences associated with various neuropathological conditions and treatments. DTI works by measuring the diffusion of water molecules in brain tissue, which can reveal important information about the integrity and connectivity of neural fibers. It has proven particularly useful in identifying early pathological changes in the brain's white matter microstructure, aiding in the study of diseases and conditions that affect neural connectivity (Alexander et al., 2007; Stephen et al., 2020). DTI's ability to assess the integrity of neural networks makes it a valuable tool in understanding cortical disconnections and the impacts of various neurological disorders such as Alzheimer, Parkinson, and other types of dementia. White matter microstructures may be altered by various risk factors, including age, severity of the dementia (Kao et al., 2019), and T2DM (Tan et al., 2016).

T2DM and AD both exert effects on the microstructural conditions within the brain and can influence cognitive functions. Despite the existing literature linking DTI measurements to AD or T2DM, the vast majority of these studies were conducted in samples comparing healthy volunteers with patient groups. To the best of our knowledge, there is no study investigating microstructural changes in white matter in relation to T2DM in AD. This study aims to conduct a comparison of DTI and clinical variables of AD patients with and without diabetes, unveiling microstructural and clinical features that contribute to an elevated of T2DM‐related dementia risk.

## 2 MATERIALS AND METHODS

### 2.1 Study population

The study involved 64 individuals diagnosed with AD, with an average age of 75.02 (SD 6.99). Among the participants, 37 patients (57.8%) were female. Out of the patients with AD, 31 individuals had T2DM. This retrospectively designed study was carried out with patients who applied to Bezmialem Vakif University Hospital between August 2015 and December 2020. Patients with T2DM and AD diagnosis were scanned from the hospital database system according to International Classification of Diseases, 10th revision (ICD‐10 codes) (T2DM and dementia in AD ICD‐10 codes, respectively: E11, F00). The patients who were diagnosed with probable AD diagnosis clinical criteria according to the National Institute of Aging‐Alzheimer's Association diagnostic criteria were included in the study by confirmed patient data. Moreover, the diagnosis of T2DM was confirmed according to American Diabetes Association criteria. Participants with severe cerebrovascular diseases (ischemic stroke and hemorrhage), trauma, tumor, mixed dementia, and with a history of psychiatric diseases (e.g., schizophrenia, major) depression were excluded. The blood laboratory results and clinical information of the patients were retrieved from the hospital database. This research received approval from the Ethics Committee of Bezmialem Vakif University (2021/389 (2)).

### 2.2 The acquisition and analysis of DTI data

Magnetic resonance imaging (MRI) examination was performed at a 1.5‐T unit (Siemens) using the standard head coil for signal perception. The DTI acquisition utilized 30 unique diffusion‐sensitive gradients with two distinct *b* values (*b* = 0 and *b* = 1000 s/mm^2^) employing a single‐shot echo‐planar sequence characterized by a TR/TE of 3300/89 ms. The imaging matrix was set to 28 × 128 (Acquisition Matrix PE: 128, Recon Matrix PE: 128), optimizing spatial resolution. The field of view was established at 230 × 230 mm^2^, ensuring comprehensive coverage. Each acquired axial slice had a thickness of 5 mm with a 5 mm inter‐slice gap, enhancing volumetric representation. The slice sampling encompassed 21 slices, capturing detailed structural information. These parameters collectively aimed to achieve high‐quality DTI, providing valuable insights into tissue microstructure and connectivity.

In the DTI preprocessing phase, we utilized DTIprep (version 1.2.4), a robust tool known for its effectiveness in quality control and artifact correction within DTI datasets. This process ensured the reliability of subsequent analyses. Following this, fractional anisotropy (FA) and mean diffusivity (MD) analyses were performed using the Tract‐Based Spatial Statistics (TBSS) pipeline of FSL FMRIBS's library (version 6.0.6). This involved registering FA images to a common space (TBSS 1), creating a mean FA image and extracting the mean FA skeleton (TBSS 2), registering individual FA images onto the mean FA skeleton (TBSS 3), and projecting MD and FA data onto the mean FA skeleton (TBSS 4).

The study cohort comprised patients diagnosed AD, categorized into two groups based on the presence or absence of comorbid T2DM. This stratification allowed for a comprehensive investigation into the influence of T2DM on DTI metrics in AD patients. The rigorous implementation of these preprocessing and analysis steps aimed to yield accurate and reliable measures of white matter microstructural alterations associated with AD, with a specific emphasis on the impact of comorbid T2DM. This approach ensures a thorough understanding of the intricate relationships between DTI metrics, neurodegenerative processes in AD, and the additional influence of T2DM on white matter microstructure.

For the fiber tracking analysis, we utilized the FSL FMRIB's Software Library (version 6.0.6) in our study for the thorough preprocessing and tractography analysis of DTI data. The process commenced with eddy current correction, specifically addressing distortions and artifacts induced by eddy currents within the DTI data. This correction plays a crucial role in enhancing the precision of diffusion measurements, ensuring the reliability and accuracy of subsequent analyses. After the eddy current correction, we utilized the “bedpost” model (Bayesian estimation of diffusion parameters obtained using sampling techniques) to model diffusion parameters. This step facilitated a refined comprehension of the brain's complex fiber architecture, with the “bedpost” model proving particularly adept at discerning crossing fibers, a crucial factor for precise tractography.

In the final phase of our preprocessing, we employed “probtrackx” a probabilistic tracking technique, to delineate the connectivity pathways of the corpus callosum, genu, and splenium, as well as other significant white matter tracts like the fornix, cingulate gyrus, external capsule, superior longitudinal fasciculus (SLF), uncinate fasciculus, and inferior fronto‐occipital fasciculus. These regions, previously identified as significant in diabetes‐related meta‐analysis (Alotaibi et al., 2021), served as seed points for mapping the entire network of fiber tracts emanating from these pivotal areas, allowing for an extensive assessment of the structural connectivity and integrity associated with the amygdala and hippocampus.

Following tractography, we subjected every “fdt_paths” image to a stringent permutation‐based analysis, executing 5000 permutations to robustly ascertain the differences between AD and T2DM cohorts. To account for multiple comparisons and control family‐wise error rates, we utilized Threshold‐Free Cluster Enhancement correction. This advanced method augmented our detection of genuine neural connectivity patterns while effectively mitigating the risk of false positives.

### 2.3 Statistics

All analyses were performed using IBM software (SPSS 28.0 for Windows; IBM Corp). Continuous variables, if normally distributed, were presented as means  ±  SDs, and two groups were compared by independent sample *t*‐test. Continuous variables, if not normally distributed, were presented as median (quartile) and compared by Mann–Whitney *U*‐tests. Categorical variables were presented as counts (percentages) and compared by Chi‐square test. Normality was evaluated by the Kolmogorov Smirnov test. The level of significance was set at ≤.05. Also, linear regression analysis with the stepwise method was used to predict the biochemical values that affect the extraction value of cross fiber in left SLF of patients with AD&T2DM.

## 3 RESULT

The 64 patients with AD involved in our study were categorized based on the presence or absence of a diagnosis of T2DM. Of these, 31 individuals had both T2DM and AD, while the remaining 33 participants had AD without T2DM. There was no significant difference between the MMSE scores of AD with and without T2DM (*p* > .05). In addition to variables related to glucose metabolism, there were significant differences between the two groups in triglyceride, Na, eGFR, urea, BUN, creatinine, eosinophils, MCH, hematocrit, hemoglobin, and MCV levels. Various clinical data of the patients are given in Table 1.

**TABLE 1:** Various clinical features of Alzheimer's patients with and without Type 2 diabetes mellitus (T2DM).

Variable	DM and AD (*n*: 31)	AD (*n*: 33)	*p*
Age	74.35 (7.55)	75.64 (6.47)	.468
Female, *n* (%)	19 (61.3)	18 (54.5)	.585
MMSE	17.23 (5.27)	18.13 (5.55)	.562
Fasting glucose (mg/dL)	130 (102–194)	98 (85.25–106)	<.001
Hemoglobin A1c (%)	6.71 (6.10–7.92)	5.55 (5.33–5.69)	<.001
LDL (mg/dL)	120.20 (94.92–171.97)	119 (104.42–153.67)	.918
HDL (mg/dL)	50.15 (11.89)	56.71 (17.54)	.236
Triglycerides (mg/dL)	145 (109–194)	85 (68–134)	.012
TSH (mIU/L)	1.06 (0.62–1.37)	1.07 (0.76–1.80)	.580
Ferritin (µg/L)	49.84 (40.41–76.54)	84.81 (38.89–152.95)	.206
Iron (µg/dL)	50 (38–74)	69 (53–104)	.096
B12 (ng/L)	342.5 (231.75–472.50)	321 (219–447)	.917
Folic acid (µg/L)	5.95 (3.97–7.52)	5.55 (4.22–7.45)	.924
25(OH)D (ng/mL)	19.35 (10.92)	21.37 (9.80)	.537
AST (U/L)	17.14 (4.56)	19.27 (6.21)	.201
ALT (U/L)	12 (10–22.5)	13 (11–19.50)	.755
Na (mEq/L)	139 (135.5–140)	141 (139–142)	.001
K (mmol/L)	4.48 (0.52)	4.44 (0.51)	.654
Ca (mg/dL)	9.38 (0.41)	9.34 (0.55)	.795
eGFR (mL/min/1.73 m^2^)	56.80 (19.73)	73.50 (14.79)	<.001
Urea (mg/dL)	43 (33.25–64.25)	34 (26.5–41.5)	.028
BUN	19.86 (14.83–31.07)	16.35 (12.26–21.26)	.077
Creatine (mg/dL)	1.13 (0.83–1.29)	0.88 (0.73–0.96)	.007
Neutrophil (10×3/µL)	4.75 (1.34)	4.72 (2.09)	.948
Lymphocyte (10×3/µL)	2.08 (0.54)	1.93 (0.59)	.294
Eosinophils (absolute number)	0.21 (0.15–0.39)	0.10 (0.07–0.15)	.001
Monocyte (absolute number)	0.55 (0.49–0.69)	0.51 (0.45–0.64)	.167
Basophil (absolute number)	0.08 (0.05–0.10)	0.06 (0.04–0.08)	.081
Leukocyte	7.74 (1.48)	7.47 (2.32)	.596
Erythrocyte	4.34 (0.59)	4.57 (0.57)	.119
Hematocrit	38.99 (34.20–40.89)	42.29 (38.8–43.93)	.001
MCH	28.7 (26.73–30.30)	29.87 (28.22–31.38)	.026
NLR	2.21 (1.94–2.97)	2.08 (1.57–3.26)	.665
PLR	112.26 (89.74–150.76)	113.71 (87.57–135.35)	.851
Hemoglobin (g/dL)	12.15 (1.72)	13.53 (1.50)	.002
Platelet (10×3/µL)	221 (182.8–288)	198.5 (179.25–264)	.166
MCV	86.20 (7.24)	90.24 (7.00)	.031

*Note*: Normally distributed continuous variables were presented as mean (SD), nonnormally distributed continuous variables were presented as median (Q1–Q3), Categorical variables were presented as counts (percentages).

Abbreviations: AD, Alzheimer's disease; eGFR, estimated glomerular filtration rate; NLR, neutrophil–lymphocyte ratio; PLR, platelet–lymphocyte ratio.

**FIGURE 1:**
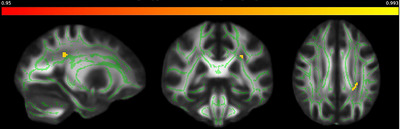
Comparative white matter fiber density in the left superior longitudinal fasciculus of Alzheimer's disease (AD) patients, with and without Type 2 diabetes mellitus (T2DM). Green area denotes the FMRIB58 skeleton (1 mm) for white matter reference, and yellow area highlights significant permutation differences in fiber density between the two patient groups.

No statistically significant differences were observed in the FA and MD TBSS analyses. However, a meticulous cross‐fiber analysis revealed a distinct pattern: The left SLF displayed notably more intense and robust fiber structures in the AD patient group without T2DM. In stark contrast, AD patients with comorbid T2DM exhibited markedly diminished fiber intensity in the same region. This discrepancy implies a significant variance in the microstructural integrity of the brain's white matter between these two patient cohorts (Figure 1). Subsequently, we extracted significant values for each patient from the cross‐fiber analysis results. This extraction aimed to facilitate a correlation analysis, exploring relationships between these values and various cognitive and blood parameters. When the correlation of extraction value and clinical data was examined, a significant correlation was detected with creatine (*p* = .038, *r* = −.278) and hematocrit (*p* = .035, *r* = .270). Linear regression was performed between extraction value and variables with *p* < .25 in correlation with extraction value. Because of the high correlation between creatinine and eGFR values, creatinine was included in the regression analysis, as it has a stronger relationship with the extraction value. Creatine (*p* = .019) and ALT (*p* = .052) according to model 2, creatine (*p* = .042) according to model 3 are clinical factors that increase the risk of diabetes‐related microvascular impairment in the SLF in patients with AD (Table 2). Model 3 shows that for each unit increase in creatinine levels, there is a slope of 84,695 units decrease in extraction values in the left SLF. This may indicate that white matter microstructure in the left SLF are sensitive to the impairment of kidney function associated with diabetes in AD patients.

**TABLE 2:** Linear regression analysis of variables associated with changes in extractions in the left superior longitudinal fasciculus.

Model		Unstandardized coefficients	Standardized coefficients	*t*	Sig.
		B	Beta		
1	(Constant)	592,528.151	–	3.252	.002
	Age	−3673.498	−.251	−1.860	.069
	HbA1c	−10,115.553	−.196	−1.492	.142
	ALT	−3486.418	−.269	−1.966	.055
	Creatinine	−99,415.628	−.341	−2.460	.018
2	(Constant)	496,825.561	–	2.877	.006
	Age	−3261.192	−.223	−1.647	.106
	ALT	−3577.193	−.276	−1.993	.052
	Creatinine	−99,583.093	−.342	−2.434	.019
3	(Constant)	228,353.872	–	3.933	<.001
	ALT	−3161.588	−.244	−1.750	.086
	Creatinine	−84,695.928	−.291	−2.087	.042

*Note*: Adjusted *R* square: Model 1 = .122, Model 2 = .100, Model 3 = .069.

## 4 DISCUSSION

This study aimed to investigate the differences between the brain white matter microstructure of AD patients with and without T2DM and the clinical data that may be associated with it. This is the first study we know of to examine white matter changes associated with the presence of T2DM in AD. In our research, we have obtained the following findings: 1—We observed a significant impairment in the left SLF among patients with AD who also have T2DM. 2—This impairment in patients with AD and T2DM can be anticipated by an elevation in creatine levels.

Patients with T2DM have an increased risk of dementia and an accelerates development of AD (Ciudin, 2016). Cross‐sectional research studies have consistently linked T2DM to global brain atrophy (van Harten et al., 2007). However, it is not clear what T2DM changes in the pathology of AD. There is limited neuroimaging literature, other than DTI, investigating the effects of T2DM in AD. Similar to our research design, in structural MRI studies comparing AD patients with and without T2DM, it was reported that patients with AD and DM had significantly higher cortical atrophy caused by nonvascular mechanisms (Biessels et al., 2006) and significantly reduced pons volume (Wang et al., 2020). This research identified a significant impairment of the left SLF in AD patients with T2DM with DTI. SLF, which passes through the frontal and parietal lobes to reach the occipital lobe, is an important tract that frequently reported white matter impairments in patients with T2DM or AD (Luo et al., 2020; Sanjari Moghaddam et al., 2019). But DTI studies in the literature have frequently compared AD and T2DM patients to healthy controls and identified various white matter alterations that may be associated with these diagnoses. Alatoibi et al. reviewed 29 DTI studies on cognitive status and microstructural changes in T2DM patients. These studies focused on comparing brains with diabetes and healthy controls. Most of the findings in these studies were reported in the frontal and temporal lobes in T2DM patients and generally reported differences between groups in axial radial diffusivity parameters, as well as regions with low FA values or high MD values (Alotaibi et al., 2021). In DTI studies involving T2DM patients, when compared to nondiabetic controls, various microstructural changes were observed in the corpus callosum, corona radiata, cingulum, uncinate fasciculus, superior longitudinal fasciculi, inferior longitudinal fasciculus, thalamus, corticospinal tract, inferior frontal–occipital fasciculus, cerebellum, internal and external capsules, fornix, and hippocampus (Kim et al., 2016; Nouwen et al., 2017; Reijmer et al., 2013; Tan et al., 2016; Xiong et al., 2019; Yau et al., 2013; Yoon et al., 2018; Zhang et al., 2014, 2016). Similar to our findings, several studies have also reported extensive decreases in microstructural integrity in the SLF white matter tracts of T2DM patients, which may be related to cognitive functions (Yau et al., 2014; Zhang et al., 2016). Numerous studies have investigated the correlations between DTI measures and cognitive functions to elucidate the neural structures contributing to cognitive deficits in T2DM (Sanjari Moghaddam et al., 2019). These investigations reveal that compromised microstructural integrity in the cingulum is linked to diminished verbal memory (Hoogenboom et al., 2014). Additionally, a decrease in white matter connectivity between the hippocampus and temporal lobe memory is associated with memory impairment (van Bussel et al., 2016). Some of the DTI studies comparing patients with AD and normal cognition reported impairment of neurofiber tracts in SLF related to AD pathology (Liu et al., 2011; Zhong et al., 2023; Zimny et al., 2012), whereas others found no significant difference (Pievani et al., 2010; Zhang et al., 2011). Upon reviewing our findings alongside the existing literature, although we cannot assert that the microstructural deterioration observed in the SLF is exclusive to diabetes pathology, it appears that diabetes‐associated deterioration in the SLF substantially amplifies the degeneration linked to AD.

Possible correlation between the endocrine and clinical variables and microstructural alterations in T2DM have been evaluated. Several studies have indicated that longer disease duration is associated with more severe microstructural defects in various regions of the brain, including right prefrontal, cerebellum, temporal lobe, right caudate, bilateral cingulate gyrus, pons, and parahippocampal gyrus as well as disrupted connectivity connections from the right superior frontal gyrus to the right cerebellar crus II (Fang et al., 2017; Hsu et al., 2012; Xie et al., 2017). In individuals with diabetes, poorer glycemic control is correlated with reduced efficiency and longer connection paths in the global brain network and it may disrupt the topological integration of the brain, potentially impacting cognitive function (Kim et al., 2016). When compared to diabetes patients without comorbidities or nondiabetic controls, diabetic patients with a comorbid characteristic have demonstrated an even greater deterioration in the gray matter/white matter integrity (Sanjari Moghaddam et al., 2019). The findings of our study suggest that impaired renal function in AD patients with T2DM is associated with microstructural changes in the SLF. These findings support the literature that diabetes‐related comorbidities cause disruption in white matter integrity.

Diabetic nephropathy, the most prevalent complication of T2DM, is caused by the microvascular complications of diabetes mellitus (DM) and is the primary cause of end‐stage renal disease globally (Thipsawat, 2021). In our study, AD patients with T2DM had more impaired renal function than patients without diabetes. Although the cognitive scores of these patients were not different from each other, there were differences in white matter integrity. Regression analyses also showed that impaired renal function increased the risk of impaired white matter integrity in left SLF in AD patients with diabetes. According to a meta‐analysis, chronic kidney failure emerges as a significant and independent somatic risk factor contributing to the development of cognitive decline. Furthermore, this association is independent of the stage of CKD but is even stronger in moderate to severe CKD (Etgen et al., 2012). In several studies, a decline in renal function has been linked to the development of dementia from various causes, excluding AD. These findings suggest that the observed association might be mediated through vascular mechanisms (Helmer et al., 2011; Seliger et al., 2004). Our findings substantiate these hypotheses. The development of AD in association with T2DM may stem from mechanisms distinct from typical AD pathology, potentially influenced by complications related to diabetes‐induced renal issues.

The current study has a few limitations. Although the sample size of our neuroimaging study is deemed reasonable, our study is limited by the inclusion of only AD patients with and without DM. Unfortunately, due to the retrospective design of this study, we encountered challenges in accessing an adequate number of non‐AD patients with T2DM within the MR records of our hospital. Although this design allowed us to investigate diabetes‐related changes in AD patients, it restricts the generalizability of our findings to other populations. Therefore, our article is limited to suggesting diabetes‐related changes in AD patients. The identification of microstructural impairments linked to T2DM could have been enhanced by the inclusion of non‐AD patients with T2DM. To provide a more comprehensive understanding of the relationship between diabetes and cognitive impairment, future studies should consider including patients with diabetes without AD and healthy controls. It is noted in the literature that antidiabetic medications and disease duration may impact cognitive functions. A limitation of our study is that our T2DM group is heterogeneous and has not been standardized in terms of drug use and disease duration.

## 5 CONCLUSION

As a consequence, cognitive impairment is now acknowledged as a complication of T2DM. Although the correlation between T2DM and AD has been documented, explicit pathologic mechanisms remain elusive. The findings of this study, comparing AD patients with and without T2DM, indicate that compromised renal function could potentially contribute to impaired white matter integrity. Subsequent research endeavors may offer greater clarity on the mechanisms through which the impact of impaired renal function on the brain becomes manifest.

## AUTHOR CONTRIBUTIONS

**Betul Sumbul Sekerci**: Conception and design; acquisition of data; analysis andinterpretation of data; drafting of the manuscript; Final approval of theversion to be published. **Halil Aziz Velioglu**: Analysis and interpretation of data; drafting of the manuscript;Final approval of the version to be published. **Abdusselam Sekerci**: Conception and design; acquisition of data; Final approval of theversion to be published.

## CONFLICT OF INTEREST STATEMENT

There are no conflicts of interest.

## FUNDING INFORMATION

None

### PEER REVIEW

The peer review history for this article is available at https://publons.com/publon/10.1002/brb3.3533.

## Data Availability

The data that support the findings of this study are available from the corresponding author upon reasonable request.
